# A Consideration of Some of the Objections Offered by Physicians to Amalgam Fillings*Read before the Rhode Island (State) Dental Society, at their meeting held in Providence, R. I., Tuesday evening, January 8th, 1895.

**Published:** 1895-03

**Authors:** Charles H. Taft

**Affiliations:** Boston, Mass.


### A CONSIDERATION OF SOME OF THE OBJECTIONS OFFERED BY PHYSICIANS TO AMALGAM FILLINGS. *

CHARLES H. TAFT, A. B., D. M. D., BOSTON, MASS.

MR. PRESIDENT AND GENTLEMEN OF THE RHODE ISLAND DENTAL SOCIETY :—For the invitation to be present upon the occasion of this your semi-annual meeting, and to present for your consideration a paper which I have already given to the profession through the medium of the *International Dental Journal*, entitled "Injurious Effects of Amalgam Fillings," permit me to offer you my cordial thanks and appreciation. But, having a natural delicacy in reading before any society a paper which had already been given to the whole profession, it occurred to me that I might be able to give you some new thoughts for a profitable discussion upon the same subject somewhat modified, a subject in which both medical and dental professions seem to be equally interested.

If I appear to have very positive thoughts or convictions upon a subject in which I have become deeply interested during the past two or three years because of the trouble I have taken to investigate the merits of the statements made by physicians, and to see whether such statements were confirmed or not by actual cases in practice, be assured it is not from any desire or purpose of making dogmatic assertions which will not admit of verification, or which rest upon no logical or scientific foundation, nor for the purpose of commending or condemning any school or system of medicine; but because we have found a firm conviction among physicians of both the two great schools of medicine that amalgam fillings often prove a great obstruction to the action of indicated remedies in the treatment and cure of the sick, and because we are often requested, either by patients themselves or by the physician in whose care such patients may

---

* Read before the Rhode Island (State) Dental Society, at their meeting held in Providence, R. I., Tuesday evening, January 8th, 1895.

be, to remove all amalgam fillings in their teeth, and to substitute some other material in the place of them.

Having met with an opposition then which commands attention, if not, indeed, respect, it becomes us to inquire into its reasonableness or unreasonableness, to either prove or disprove the statements made, to the end that we may at least be able to discuss the subject intelligently, not only among ourselves, but with our friends of the medical profession.

Many of us, no doubt, are at first nettled by what seems to be an unwarranted interference upon the part of physicians with all who are practicing our specialty in a seeming dictation as to what we shall or shall not use as suitable filling materials, and our first impulse is to ask such patients as may be sent to us for the purpose of having their amalgams removed the question, "What crank have you for a physician?" and then proceed to inform those patients that such advice is the most veritable bosh, humbug, or nonsense.

After we have relieved our minds to a certain extent by dwelling upon how little the physician really knows about amalgam fillings, or indeed about anything else pertaining to the treatment of the teeth or the diseases of the oral cavity, there comes the thought that the work of removing the fillings and substituting something else in their place is not to be done without pecuniary recompense, so we finally conclude to waive any further discussion with the patient, simply remarking, "Very well, if you are determined to have the fillings removed and something else inserted in their place, and don't mind the unnecessary expense, why, that is your affair, only I want to assure you that physicians don't know what they are talking about when they declare that amalgam fillings can ever exert any injurious effect. Dentists have been filling teeth with this material for fifty years, and I tell you what all dentists *know*, that there are no injurious effects whatever from amalgam fillings."

This, perhaps, is a fair statement of what comes into our minds to say when patients present themselves for the purpose I have stated. Such thoughts I myself have frequently given expression to in the past, for the reason that I had always been a great sceptic as to the truth of the assertion that amalgam fillings could ever possibly offer any obstruction to the action of medicines, and because I had never taken the slightest trouble to investigate the statements of physicians upon the point at issue to satisfy myself of their accuracy or inaccuracy, and it was easier, I found, to make a general denial or a dogmatic assertion to the effect that amalgam fillings never exerted any injurious effect than it was to attempt to give a reason, based upon any real scientific knowledge to give it value, why they were not. And, furthermore, I had found that amalgam, or rather copper amalgam, was too valuable a material for preserving the teeth of my patients to be lightly discarded either because of some whim of the patient or because a certain few in the medical profession did not approve of it.

But there came a time when it was a pleasure to me to take up the matter, and to investigate the statements made by physicians without prejudice, and becanse of the satisfaction which comes from being able to give a reason for one's convictions when called upon to defend them; to show the philosophy of your reasoning and the faith that is in you.

Simple assertions and denials any one can make, nor is it necessary to say they generally count for nothing, but one fact or principle evolved by inductive and deductive processes of reasoning, and plainly demonstrated by actual experiment, so there is no way of getting around its acceptance, is worth a thousand idle or thoughtless assertions from you or me, which prove nothing, having nothing but an individual opinion back of them on which to rest.

A man need never hesitate to have positive convictions or the courage to express them however much others may differ with him, or for fear of their being held up to ridicule, provided he is able to defend them with logical reasons, and while I do not come before you to make the assertion, much less to prove to you beyond all question of doubt that amalgams are *always* a source of evil; that they invariably prove a great obstruction to the action of indicated remedies in the treatment of the sick, and that they should never be used under any conditions, I do come here to say frankly that it is my belief they very often do this from what I have learned by observation and from the study of the action of drugs; and, let me say further, that I believe we should never at least refuse to comply with the instructions of the physicians when patients come to us to have their amalgams removed even though we choose to consider and to use amalgam as a valuable filling material under certain conditions.

It is probably true that the strongest protests against amalgam fillings come from the homœopathic school of medicine, but it is by no means confined entirely to that school. I have had patients come to me from physicians of the allopathic school, for whom it was claimed they could do absolutely nothing in the way of effecting permanent cures until all amalgam fillings were removed, and doubtless my experience in this respect has been but that of many of you.

What, then, are the objections offered by physicians to amalgam fillings? Briefly stated, they may be narrowed down to one principal and all-important one, and to that one objection I shall try to confine myself, and to base my arguments and statements upon as logical and scientific reasoning as I am capable of.

Starting with the premise, then, that it is the Mercury alone which is conceded by physicians in both the two great schools of medicine to be a source of evil, and which has proved to act as such an obstructive agent in many cases to the proper curative action of a given medicine, it behooves us to demonstrate in what particular way this fact is made evident, and this brings us, in the beginning, to the subject of the susceptibility of people in general to the action of drugs, medicines, poisons, or noxious influences of various kinds.

Let us take a few illustrations.

A person whose system or vital force is at any particular time peculiarly susceptible to Arsenic cannot sleep in a room the wall-paper of which contains Arsenic without acquiring symptoms of arsenical poisoning, while another person may sleep in the same room for an indefinite period and never suffer any injurious effect.

Another person, who is peculiarly susceptible to Phosphorus, may work in a match factory, or, indeed, have but to take a few inhalations from a bottle containing Phosphorus in a very high potency, to develop phosphor-necrosis of the maxillary bones or other symptoms of Phosphorus poisoning, while another person would develop no such symptoms in either case.

Still another person has but to walk through or even near a bed of poison ivy to get well-marked symptoms of ivy poisoning, while another person can walk through the bed or handle the leaves with perfect impunity.

Many people at a certain season of the year are susceptible to some peculiar force or influence in the atmosphere, and become regular victims of what is known as hay fever, with symptoms peculiar to that affliction, while others never have it.

In like manner others are peculiarly susceptible at times to the micro-organisms which are said to be productive of the so-called infectious diseases, and give unmistakable evidence that such susceptibility is not a product of their imaginations, while others suffer no ill effects from exposure to them. Similar illustrations might be cited without number.

What inferences or conclusions, then, are we to draw from such observations?

First, that there is some force existing which we can neither see, feel, taste, or smell, much less catch in our hand and place upon a glass slide, there to subject its molecules or substance, whatever it may be composed of, to the search-light of the microscope, with the hope of finding some faint evidence or trace of the crude material of the drug, poison, or germ.

No such attempt to catch and discover the hidden, inmost power of that invisible and yet unmistakable force is possible. To discover how highly potentized, or how infinitesimally small its molecules may be, is simply beyond all human power, and yet we have seen certain manifestations, or, rather, actual pathological conditions, produced in systems susceptible to such forces, attended with a train of symptoms in each instance peculiar to that drug or germ alone and to no other.

Equally manifest is it that this force has been directed, not upon the material organs or tissues of the body, but rather upon the vital force—that unseen and intangible something that constitutes the sole difference between a live man and a dead man; and, furthermore, that this vital force has been thrown out of its normal equilibrium for the time being by this drug force, as shown by the lack of harmony among the various organs or tissues of the body in properly performing their functions.

These illustrations, it seems to me, should be sufficient to make us realize that it can never be a question of how many grains or drachms or ounces of any drug or poison it may be necessary to give either in the treatment of disease, or how many grains or ounces it may require to throw the vital force out of its normal equilibrium, and to produce a want of harmony among the organs and tissues in properly performing their functions; for, were that the case, on the ground that a tenth of a grain of a given drug was a good curative dose, a half-grain ought to be a still better one; and if a half-grain is a good thing, a whole grain is still better; and if a whole grain, why not an ounce? and if an ounce, why not a pound, and so on *ad infinitum?*

Upon this same line of reasoning, a large dose of the crude material should be much more effectual in producing disease than one so small that its molecules could be neither discovered nor measured, where all that was left of it, in fact, was an invisible yet unmistakable force.

Rather, gentlemen, must we recognize and admit the fact that, when the vital force has left the body, the material organs and tissues have no further use for drugs; for, if drugs are given for the purpose of exerting their action upon the material organs and tissues themselves, why should they not prove just as effectual after the vital force is no longer there to animate them?

Does it not stand to reason, then, that this vital force is, what Hahnemann affirmed it to be, a spirit-like force, and which, when becoming diseased, must be treated or met with a similar spirit-like or dynamic force rather than with a crude material one?

What else can such forces as I have already referred to be if *not* dynamic in their very nature? How else can we explain either their nature or account for their action? for, surely, they cannot be said to be directed effectually upon the organs and tissues of the body themselves in a live man any more than they can make their influence felt upon the same organs and tissues in a dead man.

Keeping in mind, then, this fact, that it is the vital force which must always be met with a similar dynamic force when a drug is given for its curative effect in restoring harmony among the tissues and equilibrium to the force which governs them, let us try to discover what its nature must be, though the scalpel, the microscope, or a dozen other agents or means that we might employ would never help us to catch and examine it.

In the study of embryology and zoology the miscroscope has given us visible demonstration of the fact that all life is cell-life, and that such life is manifested in many of the lowest forms of the vegetable and animal kingdoms by the existence of but a single cell. Conversely, the study of pathological anatomy, which is but a manifestation of perverted life, or what we call disease, has taught us that all disease is cell disease. The human body being made up of multitudes of cells, and millions upon millions of them being so minute as to be well-nigh invisible with the highest powers of the microscope, it is clear that a medicine, in order to reach and affect them, must be equally minute; for, in order to reach them, it must enter them, and to accomplish this it must be smaller than they.

A well-known writer has said that "food, to be appropriated by the body must go to the stomach and be digested, pass from the stomach and be assimilated, but that medicine, to be effectual, should not travel this route; digestion would destroy it. It should be so minutely divided that the open-mouthed absorbents swallow it as soon as it comes in contact with the mucous membrane of the mouth. Thus unchanged it enters the circulation.

"A group of cells in a remote corner of the anatomy are hurt and crying out for help. Help is on the road—over the trunk lines—past the way-stations, out on the local road, recognized by every road official, *en route* and hurried unerringly to its destination. No need, then, of further concern or anxiety. If the doctor has selected well the drug will reach its destination."

Now, before touching upon the action of drugs and the only correct and scientific way, as I believe, of studying this action, let us, before leaving the consideration of this vital force and those unseen forces which we have shown can so easily throw it out of its normal equilibrium, call to mind the doctrine of the conservation of energy, which declares that force can never be destroyed whatever its changes in manifestation may be, as having an important bearing upon the subject we are considering, and after we have learned how to study the action of drugs, scientifically, because of studying such action in accordance with a well-established law of cure, and have also studied carefully the laws which govern this action, we shall then be able to recognize that this physical law is no less applicable to drugs and their dynamic action, even though the force is shut up in an amalgam filling and apparently inert, than it is to a simple piece of charcoal, which needs but a spark to make more manifest the hidden energy stored up in its component parts, and upon the liberation of which may show itself subsequently in manifold form, such as heat, vapor, gases, and all the compounds of both organic and of inorganic life.

Unless we thoroughly understand and accept this doctrine as one of the established laws of the universe, we shall not be likely to understand the action, or, in other words, the energy of drugs when potentized and the laws which govern it, for the energy which lies hidden in the mercury of an amalgam filling is none the less potent and subject to these same laws than it is either in its crude state or as a component part of some compound other than an amalgam filling.

Coming, then, to the study of the action of drugs, the inherent energy or force within them when potentized, how best shall such study be made? Having shown conclusively how persons in perfect health may show a well-marked susceptibility to the influence of certain drugs, poisons, or other noxious influences, let us take some one drug—Arsenic, if we please—and potentize it; that is to say, take a particle in its crude form, and develop its potentiality, or rather its dynamic force, through a continuous division and subdivision of its molecules until this force is made apparent, and we are prepared to answer the assertion of the sceptic—that there is no more medicinal effect to this force in the form in which we have it safely bottled up than there would be in a pail of pure spring-water, by an actual test of this force upon the healthy human subject.

Continuing the process of the subdivision of these molecules we shall eventually reach a point when the microscope will no more reveal a single molecule or trace of the crude material of the Arsenic than it would if we could catch the molecules of the same drug or force of that drug which poisons a person who sleeps in a room whose wall paper contains Arsenic, and try to discover those molecules or single molecule with the microscope's highest powers. And yet there is still a molecule of Arsenic left of our original particle that we started with, for who would wish to assert that when a molecule has been divided and subdivided for an infinite number of times there has been a limit reached to its further subdivision? Are there any boundary laws by which Infinity may be measured? No, not one.

Being in a position where we can now assert with a considerable degree of positiveness that we have evolved or set free the dynamic force of the Arsenic, or developed, in other words, its real medicinal properties, let us prove this fact or assertion; for bear in mind we are not to accept the assertion with that "happy blind faith of childhood," nor because somebody says it is so. If there is any force there we shall be able to prove it. In what way? By giving a very small quantity of it to persons who are in perfect health. Those who are at all susceptible to Arsenic will get what are called provings of the drug, or in other words, symptoms which are peculiarily characteristic of that drug and of no other. Those who are not susceptible to it will not be affected by it, in other words, will develop no unpleasant symptoms simulating distinct pathological conditions. By proving drugs, as it is called, upon the healthy human subject, we find that each one has the power of producing symptoms characteristic of that drug and different from those produced by other drugs.

Now, as regards Mercury, it has been found that this is not only one of the most poisonous, but one of the most deep-seated and deep-acting of all drugs. Not only this, but it is one of the most difficult to eliminate from a system that has once been susceptible to its toxic effects. Indeed, its physiological effects in patients often come under our observation within the field of our own special work. It is a powerful antidote to various drugs, and it is because of its antidotal action that it proves such a powerful obstruction to the action of such medicines as the physician finds it necessary to prescribe.

Now, let us suppose a patient with some chronic affection, which has failed to yield to any kind of previous treatment, consults a homœopathic physician, for example ; and by this, I mean one who, in the practice of his art, adheres rigidly and strictly to the law which lies at the very foundation of Homœopathy. He makes a careful diagnosis of the case from the symptoms present, and makes an equally careful selection of the medicine which is found to reach the totality of such symptoms, and which should promptly give evidence of its curative action. The vital force, it may be, responds quickly to the action or force inherent in the remedy, but its effect is only temporary, and the patient soon drops back again to his former condition. Other remedies are given as the symptoms change, with no better result, and at best the patient's condition is never more than temporarily improved. The physician is puzzled and baffled in his efforts to find the right medicine which shall produce a permanent curative action. He studies the case again very carefully. Likewise the remedy. It meets the totality of all the symptoms, and *ought* to cure his patient, but it doesn't, and why? because he knows there is something which is obstructing the action of the medicine, and he looks about to find what that obstruction is. He finds the teeth of his patient have several amalgam fillings in them. He knows that Mercury is a powerful antidote to the drugs he has been using, and it must be gotten rid of before his patient can be cured. He therefore orders their removal; the dentist calls him a crank for suggesting such a thing, but what is the result of their removal? The medicines which failed to act, now have their proper curative action; the patient is quick to respond to them, and both acute and chronic conditions or affections are made to yield easily to treatment in consequence of the removal of the obstruction.

This is the whole philosophy in a nutshell, gentlemen, of the reason why our most accurate and careful prescribers of drugs in the medical profession are so much opposed to amalgam fillings on general principles.

These men are the real thinkers, the philosophers, the men of science! Men who, like Samuel Hahnemann, one of the greatest physicians and scientists the world has ever known, argue:

"When we have to do with an art, one of whose ends is the saving of human life, any neglect to make ourselves thorough masters of it becomes a crime."

And, arguing thus, leave no stone unturned to find the reason why their medicines often fail to do what is expected of them to do, recognizing and admitting the fact that if the art of medicine is founded upon a well-established law, and is, therefore, necessarily a science, the law itself can never fail, though the physician may fail in the application of it.

And yet we, as a profession, are inclined to ridicule and pooh-pooh the objection made by physicians to amalgam fillings without making any investigation into the merits of the objection, or even attempting to offer a reason why they may not act as a powerful obstruction to the action of indicated medicines, simply because we have found it serves a valuable purpose as a filling material.

Our work is of a specialized character, and is confined almost exclusively to the teeth and contiguous tissues. Beyond that we are not inclined to go. The treatment of disease has no especial interest to us, excepting in so far as it is confined to our legitimate specialty. With the action of drugs, their properties, and method of evolving the dynamic force contained therein, we have no concern. It is much easier, and requires much less waste of brain tissue, to assert that we have never known any one who had been poisoned from having amalgam fillings in his teeth. How absurd to think they could be!

We, as dentists, approve of amalgam without taking the trouble to look into the merits of a disputed question, and that is sufficient. What do a few physicians know any more than we do about its injurious effects? Let us not be dictated to by the medical profession or a few cranks upon this subject. Let them mind their own business; we will attend to ours.

These reflections, gentlemen, express, I think, quite fairly the temper of our own profession upon the attitude which has been taken upon the subject under consideration. Let us, however, bear in mind the fact that it is an opposition which has come to stay, which cannot be simply ridiculed and then laughed away. It is bound to make its presence more and more manifest in the years that are to come—a prophecy I have no hesitation of going upon record in making—and we may as well make up our minds, now that the issue has been forced upon us, that the best way, the honorable way, and the manly way, is to meet the medical profession fairly and squarely, and to discuss the question with them upon friendly terms of equality as scientific men who are practicing our profession, as they are practicing theirs, with the single purpose of finding out the truth in all things for the truth's sake, and never hesitating to proclaim or to promulgate it when found.

The essence of all true progress in any line of study or work of investigation comes largely through the efforts and the enthusiasm of men who are engaged in such work, and as a result also of that interchange of thought among men each of whom may be working upon the same subject, though along different lines, but each one always keeping the same end or object in view.

To sum up, then, the least it appears to me that we as dentists can do in order to get at the truth of the point at issue and to discuss the question intelligently with our friends of the medical fraternity is:

First. To study the action not only of Mercury but of other drugs as well both as to their toxic and their medicinal properties.

Second. To study carefully the laws which govern such action.

Third. To take a potentized drug and prove it upon one's self, or upon others, with a view to becoming satisfied whether or not there is that dynamic action or energy in them and possessing actual curative properties, which all who have faithfully studied them know they actually do possess.

Fourth. The ascertaining of what constitutes the vital force, and whether it is this force or the material organs and tissues of the body upon which the action of medicines is to be directed.

Fifth. A study of how this vital force may be thrown out of normal equilibrium; in other words, its susceptibility to drugs or other harmful influences, whether in health or disease.

Sixth. The bearing of these discoveries upon the question we are considering.

My own investigations and observations from actual cases in practice have led me to conclude that the objections offered by physicians to amalgam fillings are founded both upon common reason and upon common sense. They have taught me never to refuse to comply with the instructions of physicians to remove amalgam fillings when so requested to do. They have taught me never to make dogmatic assertions or denials which did not admit of verification as to their accuracy, and finally they have given me that enthusiasm for scientific study which comes from an earnest effort and desire to take up a subject, and to investigate it thoroughly and patiently with the single purpose of finding the truth.

There is much I would like to say still further upon the subject were it not that I have taxed too severely, perhaps already, your time and patience, but I shall be most happy, so far as I am able, to answer any questions that may occur to you to ask, and to make the discussion one not without profit to us all.

